# Differential diagnosis of adrenal adenomas and metastases using spectral parameters in dual-layer detector spectral CT

**DOI:** 10.1007/s00432-023-04931-8

**Published:** 2023-06-06

**Authors:** Lei-di Wu, Xiao-fei Yue, Lin-xia Wu, Ming Yang, Yan Chen, Jie Yu, Nan Diao, Xiao-hui Zhang, Liang-ru Zhu, Ping Han

**Affiliations:** 1 Department of Radiology, Union Hospital, Tongji Medical College, Huazhong University of Science and Technology, Wuhan, China; 2 Department of Radiology, Zhongshan City People’s Hospital, Zhongshan, China; 3 Clinical Science, Philips Healthcare, Shanghai, China; 4 Division of Gastroenterology, Union Hospital, Tongji Medical College, Huazhong University of Science and Technology, Wuhan, China

**Keywords:** Computed tomography, Dual-layer detector spectral CT, Spectral parameters, Adrenal adenomas, Adrenal metastases

## Abstract

**Objective:**

To assess the diagnostic value of spectral parameters in differentiating adrenal adenomas from metastases based on dual-layer detector spectral CT (DLSCT).

**Materials and methods:**

Patients with adenomas or metastases who underwent enhanced DLSCT of the adrenals were enrolled. The CT values of virtual non-contrast images (CT_VNC_), iodine density (ID) values, and Z-effective (Z-eff) values, the normalized iodine density (NID) values, slopes of spectral HU curves (s-SHC), and iodine-to-CT_VNC_ ratios of the tumors were measured in each phase. Receiver operating characteristic (ROC) curves were used to compare the diagnostic values.

**Results:**

Ninety-nine patients with 106 adrenal lesions (63 adenomas, 43 metastases) were included. In the venous phase, all spectral parameters were significantly different between adenomas and metastases (all *p* < 0.05). The combined spectral parameters showed a better diagnostic performance in the venous phase than in other phase (*p* < 0.05). The iodine-to-CT_VNC_ value had a larger area under the ROC curve (AUC) than the other spectral parameters in the differential diagnosis of adenomas and metastases, with a diagnostic sensitivity and specificity of 74.4% and 91.9%, respectively. In the differential diagnosis of lipid-rich adenomas, lipid-poor adenomas and metastases, the CT_VNC_ value and s-SHC value also had a larger AUC than the other spectral parameters, with a diagnostic sensitivity of 97.7%, 79.1% and specificity of 91.2%, 93.1%, respectively.

**Conclusion:**

On DLSCT, the combined spectral parameters in the venous phase could help better distinguish adrenal adenomas from metastases. The iodine-to-CT_VNC_, CT_VNC_ and s-SHC values had the highest AUC values in differentiating adenomas, lipid-rich adenomas and lipid-poor adenomas from metastases, respectively.

**Supplementary Information:**

The online version contains supplementary material available at 10.1007/s00432-023-04931-8.

## Introduction

Adrenal incidentalomas are found in 3 to 7% of adults on computed tomography (CT) images, of which nonfunctioning benign adenomas are the most common (Mayo-Smith et al. 2001; Bovio et al. 2006; Young 2007). The incidence of adrenal incidentalomas on CT images is 0.5% in patients aged 20–29 years, while the rate among individuals aged over 70 years is approximately 7% (Mayo-Smith et al. 2017; Schieda and Siegelman 2017). Metastatic diseases are also commonly seen in the adrenals. Previous studies (Young 2007) proposed that approximately 27% of patients with extra-adrenal primary malignant tumors have microscopic adrenal metastases. When no other organic metastases are found in patients with malignant tumors, accurately evaluating the biological behavior of adrenal incidentalomas has great significance for clinical treatment and prognosis.

Differences in intracellular lipids and blood perfusion are mostly used to distinguish benign and malignant adrenal tumors (Boland et al. 1998; Caoili et al. 2002; Korobkin et al. 1998). About 70% of adenomas can be diagnosed as lipid-rich adenoma according to attenuation less than 10 HU on unenhanced images, while lipid-poor adenomas (> 10 HU) could be diagnosed based on wash-out ratios (Boland et al. 1998; Caoili et al. 2002). Absolute percentage of enhancement washout (APW) ≥ 60% or relative percentage of enhancement washout (RPW) ≥ 40% are one of the diagnostic standards of adenomas (Korobkin et al. 1998; Kebapci et al. 2003). Additional examinations, such as contrast-enhanced CT, magnetic resonance imaging (MRI), positron emission tomography-CT (PET-CT) and even biopsy, may be required to differentiate atypical adrenal tumors, increasing both the economic and psychological burden of the patients (Park et al. 2016; Elbanan et al. 2020).

At present, dual-energy CT has attracted public attention for its ability to analyze the material composition and quantify contrast accumulation (Kim et al. 2013; McCollough et al. 2015; Megibow et al. 2018; Fulton and Rajiah 2018; Rassouli et al. 2017; Hojjati et al. 2017). Dual-layer detector spectral CT (DLSCT) is one of the most commonly used dual-energy CT imaging systems clinically and can generate reliable spectral parameters including virtual non-contrast (VNC) images, virtual monoenergetic images (VMI), iodine density (ID) images, Z-effective (Z-eff) images, and so on (McCollough et al. 2015; Megibow et al. 2018; Fulton and Rajiah 2018; Rassouli et al. 2017; Hojjati et al. 2017). Several previous studies have pointed out that spectral parameters such as VNC and ID based on dual-energy CT play a certain role in the differential diagnosis of adrenal tumors. A study based on DLSCT has proposed that combined VNC and ID during the venous phase enabled accurate differentiation between adrenal adenomas and metastases. However, spectral parameters of different kinds of adenomas and different phase at enhanced CT scan and their diagnostic values have yet to be explored.

In this study, the diagnostic values of various spectral parameters of DLSCT were assessed for differentiating adrenal adenomas from metastases.

## Materials and methods

This retrospective study was approved by the Institutional Review Board (IRB) of Tongji Medical College of Huazhong University of Science and Technology.

### Study participants

Patients who underwent plain CT with biphasic or triphasic enhanced DLSCT scanning of the adrenals from December 2019 to June 2021 were enrolled. The inclusion criteria were pathologically confirmed adrenal adenomas, imaging evidence suggesting newly found adrenal metastases during a 6-month follow-up period (Mayo-Smith et al. 2017), and a lesion diameter larger than 1 cm. The exclusion criteria consisted of contraindications to enhanced CT examinations, deviation from scanning protocol, poor image quality with heavy artifacts, and incomplete clinical and imaging information.

### Image acquisition

A Philips iQon Spectral CT scanner (Philips Healthcare, Best, the Netherlands) was used to examine all participants in the supine position. The scanning range was from the lower edge of the 11th thoracic vertebra to the 1st–2nd lumbar vertebra. The scanning parameters were as follows: tube voltage, 120 kV; tube current, 121–373 mAs; collimator width, 64 × 0.625 mm; rotation time, 0.5 s; pitch, 0.8; and matrix, 512 × 512. The contrast agent (iodine dose, 1.2 ml/kg; iodixanol 320 mg/ml, Hengrui Medical, Jiangsu, China) was injected through a cubital vein at a rate of 2.5 ml/s. Then, 30 ml of normal saline was administered at the same rate. Arterial phase scanning was started 8 s after the abdominal aorta attenuation reached 100 HU. The venous phase scan was started 24 s after the arterial phase, while the delayed phase scan was started 180 s after the contrast agent injection. The contrast administration protocol was the same for every examination.

The images were reconstructed with a 1 mm slice thickness and 0.8 mm interval in the axial, sagittal and coronal planes. Conventional 120 kVP poly-energetic images generated by an iterative reconstruction algorithm (iDose 4, level 3; Philips Healthcare) were uploaded to the picture archiving and communication system (PACS), while the other spectral-based imaging datasets generated in the Philips ISP postprocessing workstation (iDose 4, level 3; Philips Healthcare) were used to obtain VNC, VMI, ID, and Z-eff images.

### Image analysis

Two radiologists with 4 years (LD Wu) and 6 years (XF Yue) of clinical experience independently evaluated imaging manifestations and spectral parameters of adrenal tumors. The imaging manifestations including the morphology, margin, and enhancement pattern were observed. The region of interest was placed in the lesion covering the largest area at the section level of maximum diameter. Apparent bleeding, necrosis or cystic degeneration and calcification areas were avoided. The shape, size, and location of each region of interest remained constant across all image series. All measurements were repeated three times and the mean values were adopted for analysis. The CT values of conventional images, virtual non-contrast images (known as CT_VNC_ values), ID and Z-eff values of the tumors were measured in each phase. APW and RPW were calculated (Ng et al. 2018; Liu et al. 2019) (formula 1, 2). The normalized iodine density (NID) values and slopes of the 40–100-keV spectral HU curves (s-SHCs) values of the tumors were calculated as well (formula 3, 4). After excluding cases with negative CT_VNC_ values, the iodine-to-CT_VNC_ ratio was calculated (Nagayama et al. 2020) (formula 5).

1 $$\text{APW }= \, (\text{CT values on venous phase}-\text{ CT values on delayed phase}) \times 100\, {\% }\div (\text{CT values on venous phase}-\text{ CT values on unenhanced phase})$$

2 $$\text{RPW }= (\text{CT values on venous phase}-\text{ CT values on delayed phase}) \times 100 \, {\% }\div \text{ CT values on venous phase}$$

3 $$\text{NID }=\text{ ID tumor }\div \text{ ID abdominal aorta}$$

4 $$\text{s-SHC }= (\text{CT}40\,\text{KeV}-\text{ CT}100\, \text{KeV}) \div 60$$

5 $$\text{iodine-to-CTVNC }=\text{ ID }\div \text{ CTVNC }\times 100$$

### Statistical analysis

SPSS software (v26.0, IBM Corp, New York, USA) and MedCalc software (v19.6, Mariakerke, Belgium) were used for statistical analysis. Continuous variables were expressed as the mean ± standard deviation (*X* ± SD), and the Kolmogorov‒Smirnov test was performed to evaluate the normality of the quantitative parameters. Interobserver reliabilities of spectral parameters were analyzed by intraclass correlation coefficient (ICC) values (two-way mixed effect mode, consistency definition, single-rater type). ICC values less than 0.40 are indicative of poor reliability, 0.40–0.59 indicate fair reliability, 0.60–0.74 indicate good reliability, 0.75–1.00 indicate excellent reliability (Barth et al. 2017). The CT values, wash-out values, and spectral parameters of adenomas and metastases in each phase were compared by independent samples t test or the Wilcoxon rank sum test. *p* < 0.05 was considered statistically significant. Receiver operating characteristic (ROC) curves were generated to assess the diagnostic values of the CT values, wash-out values, and combined (CT_VNC_ value + ID value + s-SHC value + Z-eff value) and independent spectral parameters for adenomas and metastases. The area under the ROC curve (AUC), Youden’s index, sensitivity and specificity were evaluated.

## Results

### Patient characteristics

Ninety-nine patients with 106 lesions were eventually included in this study, 43 had biphasic enhanced DLSCT scanning, 56 had triphasic enhanced DLSCT scanning. Sixty-three patients (50 ± 12 years, 26 males and 37 females) had 63 adrenal adenoms and 36 patients (62 ± 8 years, 28 males and 8 females) had 43 adrenal metastases. In the patients with adenomas, there were 34 lipid-rich adenomas and 29 lipid-poor adenomas, all were unilateral including 47 lesions on the left side and 16 lesions on the right side. In the patients with metastases, 7 cases with 14 lesions were bilateral, 29 cases with 29 lesions were unilateral including 21 on the left sides and 8 on the right sides. All adrenal metastases were newly developed in patients with a history of primary malignant tumors. The primary tumors of the metastasis group included lung cancer (20 cases, 55.6%), hepatocellular carcinoma (12 cases, 33.3%), pancreatic cancer (2 cases, 5.5%), esophageal cancer (1 case, 2.8%) and lymphoma (1 case, 2.8%). Patient characteristics are shown in Table 1.

**Table 1:** Patient characteristics

Characteristic	Total (*n* = 99)	Adenomas group (*n* = 63)	Metastasis group^#^ (*n* = 36)	*p* value
Patient characteristics				
Age (years)	54 ± 13	50 ± 12	62 ± 8	< 0.05
No. of males	54 (55)	29 (46)	28 (78)	< 0.05
Body mass index (kg/m^2^)	24 ± 3	25 ± 4	23 ± 3	< 0.05
Adrenal lesions	106	63	43	–
Bilateral	7	0	7	–
Unilateral	92	63	29	–
Left	68 (74)	47 (75)	21 (58)	> 0.05
Tumor maximun diameter (mm)	26 ± 13	21 ± 8	32 ± 16	< 0.05
Lipid-rich adenomas	–	34 (54)	–	–

Data are mean ± standard deviation or number of patients, with percentages of parentheses

^#^Lung cancer (*n* = 20), hepatocellular carcinoma (*n* = 12), pancreatic cancer (*n* = 2), esophageal cancer (*n* = 1) and lymphoma (*n* = 1)

### Imaging manifestations of adrenal adenomas and metastases

**Fig. 1:**
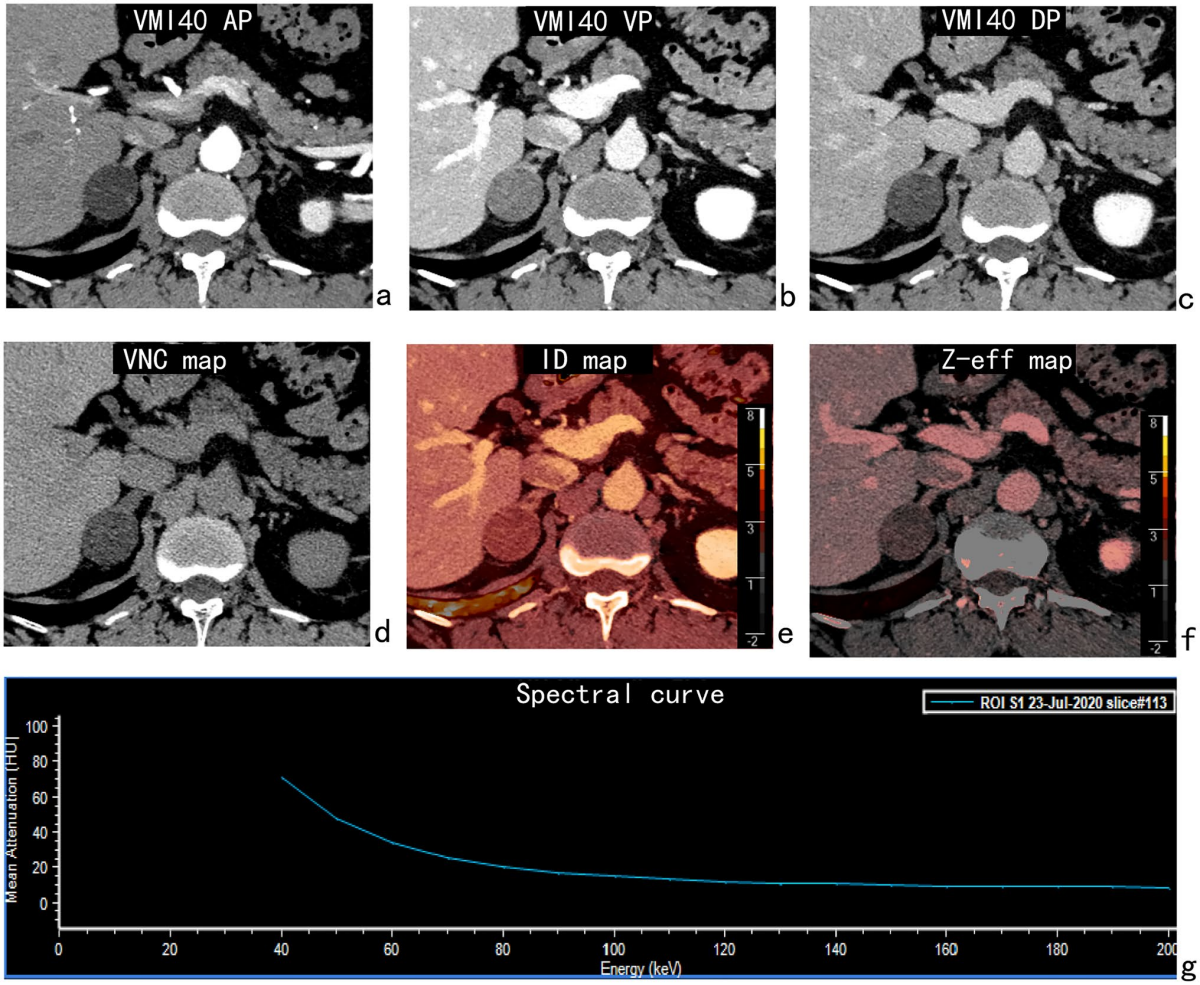
A 50-year-old man with a lipid-rich adenoma in the right adrenal gland. The 40-keV virtual monoenergetic images (VMI40) showed an oval-shaped lesion with a clear border. The CT value of virtual non-contrast images (VNC), iodine density (ID) value and Z-effective (Z-eff) value of the lesion in the venous phase were − 4 HU, 1.15 mg/mL and 8.00, respectively. The normalized iodine density (NID) and the slope of the 40–100-keV spectral HU curve (s-SHC) for the lesion in the venous phase were 0.29 and 1.43, respectively

**Fig. 2:**
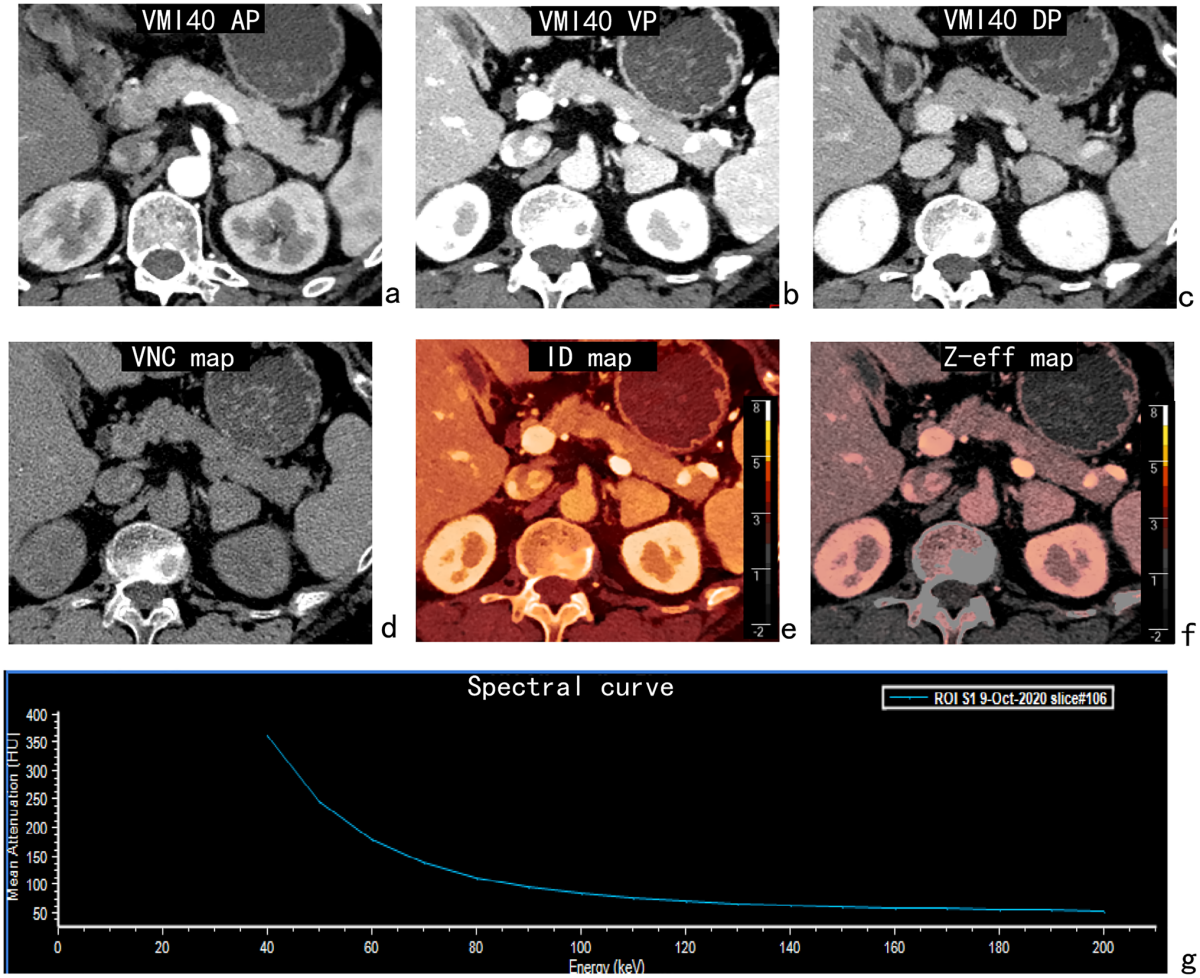
A 57-year-old woman with a lipid-poor adenoma in the left adrenal gland. The 40-keV virtual monoenergetic images (VMI40) showed a triangular lesion. The CT value of virtual non-contrast images (VNC), iodine density (ID) and Z-effective (Z-eff) value of the lesion in the venous phase were 44 HU, 3.71 mg/mL and 9.10, respectively. The normalized iodine density (NID) and the slope of the 40–100 keV spectral HU curve (s-SHC) for the lesion in the venous phase were 0.80 and 4.60, respectively

**Fig. 3:**
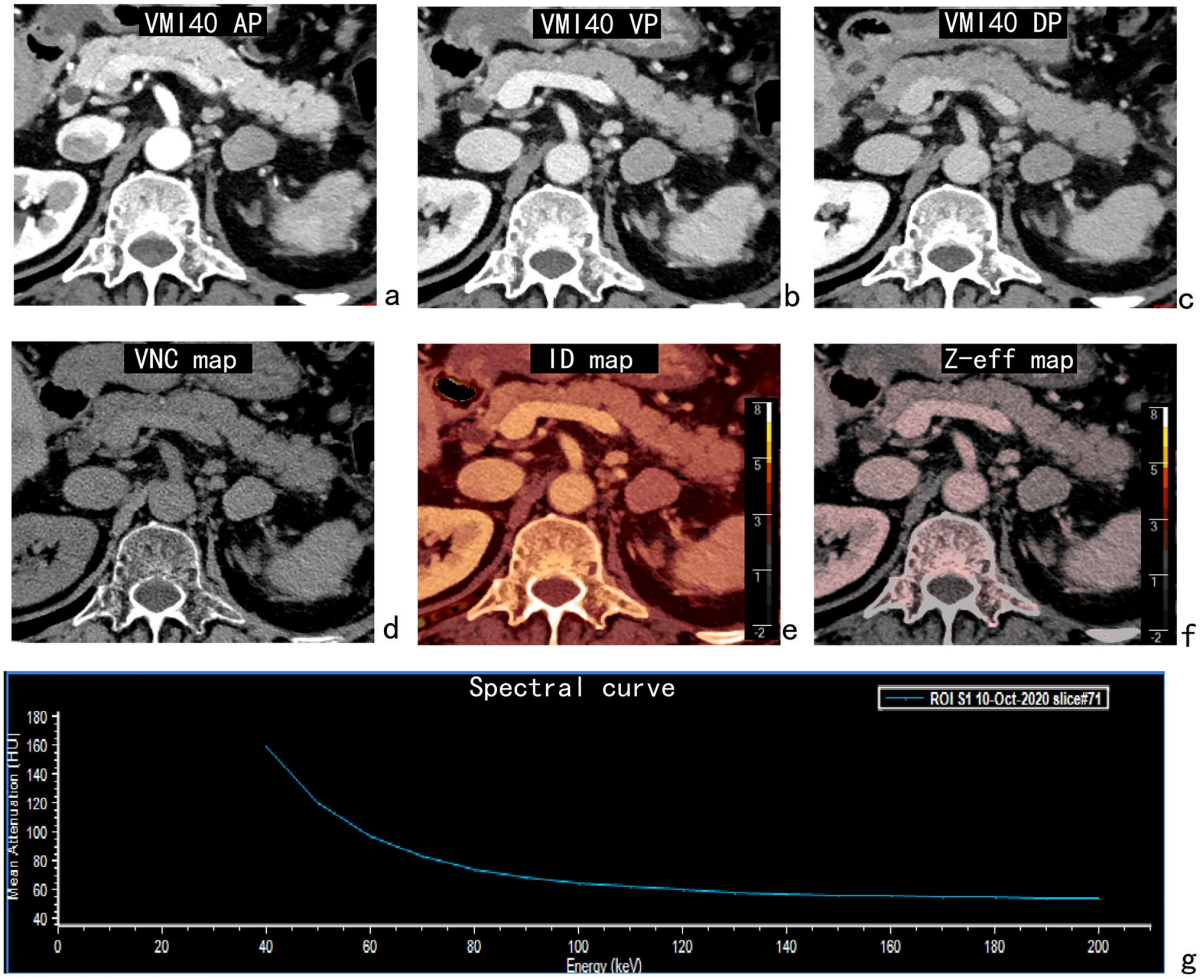
A 59-year-old man with primary hepatocellular carcinoma accompanied by left adrenal metastasis. The 40-keV virtual monoenergetic images (VMI40) showed an irregularly shaped lesion. The CT value of virtual non-contrast (VNC) images, iodine density (ID) and Z-effective (Z-eff) value of the lesion in the venous phase were 46 HU, 1.27 mg/mL and 8.04, respectively. The normalized iodine density (NID) and the slope of the 40–100 keV spectral HU (s-SHC) curve for the lesion in the venous phase were 0.36 and 1.57, respectively

Generally, lipid-rich adenomas were round or oval, homogeneous, and hypodense with clear borders, and necrosis and cystic degeneration were rarely seen. 40-keV virtual monoenergetic images (known as VMI40) showed that lipid-rich adenomas had progressive enhancement in the arterial and venous phases and washout in the delayed phase. The morphology of lipid-poor adenomas was similar to that of lipid-rich adenomas, but the attenuation was higher for lipid-poor adenomas. VMI40 showed fast enhancement of lipid-poor adenomas in the arterial phase, which peaked in the venous phase, with a rapid washout in the delayed phase. Adrenal metastases were bilateral or unilateral, lobulated or nodular and heterogeneous, presenting as ill-defined solid masses with possible necrosis, cystic degeneration and hemorrhage. VMI40 showed that adrenal metastases had significant enhancement in the arterial phase and continuous enhancement in the delayed phase. The imaging manifestations of adrenal adenomas and metastases on VMI40 are shown in Figs. 1, 2, 3).

### Evaluation of CT values, wash-out values of adrenal adenomas and metastases

The CT values, wash-out values of adenomas and metastases are shown in Supplementary Table 1. The CT values on the unehanced phase and delayed phase, APW and RPW of adenoms were significantly different from those of metastases (all *p* < 0.05). The CT values on each phase and wash-out values of lipid-rich adenomas were significantly different from those of metastases (all *p* < 0.05). The CT values of unenhanced, arterial and venous phase and RPW of lipid-poor adenomas were significantly different from those of metastases (all *p* < 0.05).

In the differential diagnosis of adenomas, lipid-rich adenomas from metastases, the AUC of the CT values on the unenhanced phase was greater than those of other CT values and wash-out values (all *p* < 0.05). The diagnostic performance of the CT values on the venous phase was greater than other the CT values and wash-out values in differanting lipid-poor adenomas from metastases (*p* < 0.05), with an AUC of 0.830 (95% CI 0.848–0.978), a sensitivity of 75.9%, and specificity of 76.7%. The AUC, sensitivity and specificity values for the CT values and wash-out values in the differential diagnosis of adenomas and metastases are shown in Supplementary Table 2.

### Spectral parameters of adrenal adenomas and metastases

Interobserver reliabilities of spectral parameters are excellent with ICC values ranging from 0.869 to 0.991. The ICC values in measuring spectral parameters of adenomas and metastases are shown in Supplementary Table 3. The spectral parameters of adenomas and metastases in each phase are shown in Table 2. In the arterial phase, the CT_VNC_ values of adenomas were significantly lower than those of metastases (*p* < 0.001). In the venous phase, all spectral parameters significantly differed between adenomas and metastases (all *p* < 0.05). Except for the iodine-to-CT_VNC_ ratio, the other spectral parameters of adenomas were significantly different from those of metastases in the delayed phase (all *p* < 0.05).

**Table 2:** Spectral parameters of adrenal adenomas and metastases in the arterial, venous and delayed phases

Spectral parameters	AP		VP		DP	
	Adenomas (*N* = 63)	Metastases (*N* = 43)	Adenomas (*N* = 63)	Metastases (*N* = 43)	Adenomas (*N* = 30)	Metastases (*N* = 32)
CT_VNC_(HU)	23.33 ± 14.29*	34.18 ± 7.48	23.85 ± 13.88*	35.38 ± 6.05	23.73 ± 14.98*	35.30 ± 5.65
s-SHC	1.32 ± 1.03	1.18 ± 0.88	2.16 ± 1.16*	1.31 ± 0.63	1.03 ± 0.68*	1.40 ± 0.58
Z-eff	7.88 ± 0.45	7.78 ± 0.37	8.24 ± 0.42*	7.95 ± 0.29	7.76 ± 0.33*	7.92 ± 0.21
ID (mg/ml)	1.08 ± 0.81	0.90 ± 0.76	1.75 ± 0.93*	1.16 ± 0.55	0.84 ± 0.55*	1.08 ± 0.38
NID	0.12 ± 0.12	0.12 ± 0.13	0.44 ± 0.21*	0.31 ± 0.14	0.32 ± 0.19*	0.45 ± 0.16
ID/CT_VNC_	11.60 ± 12.16	11.19 ± 13.17	9.74 ± 9.99*	3.33 ± 1.62	3.96 ± 1.98	3.11 ± 1.08

Note: * represents a significant difference in spectral parameters between adenomas and metastases (*p* < 0.05). Abbr: arterial phase (AP), venous phase (VP), delayed phase (DP), CT value of virtual non-contrast images (CT_VNC_), slope of spectral HU curve (s-SHC), Z-effective (Z-eff), iodine density (ID), normalized iodine density (NID), and iodine-to-CT_VNC_ (ID/CT_VNC_)

**Fig. 4:**
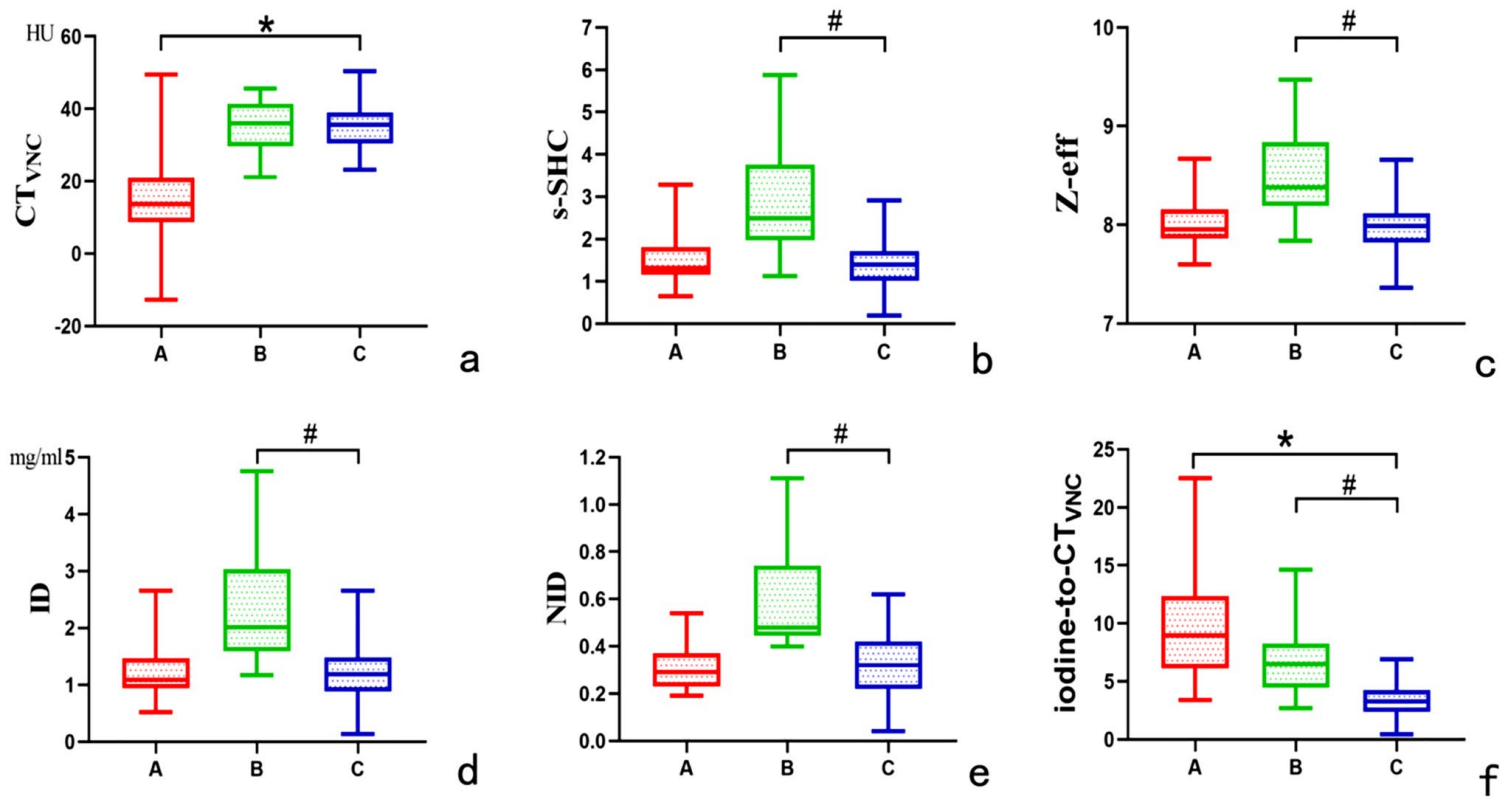
Box and whisker plots showing the spectral parameters of lipid-rich adenomas (A group), lipid-poor adenomas (B group) and metastases (C group) in the venous phase. The centerline and height of each box represent the median value and interquartile range, respectively. **a** The CT value of virtual non-contrast images (CT_VNC_ value) of lipid-rich adenomas was lower than that of metastases (14.18 ± 10.33 HU vs. 35.38 ± 6.05 HU). **b**–**e** The slope of the 40–100-keV spectral HU curve (s-SHC), Z-effective (Z-eff) value, iodine density (ID) and normalized iodine density (NID) of lipid-poor adenomas were higher than those of metastases (2.91 ± 1.20 vs. 1.31 ± 0.63; 8.52 ± 0.41 vs. 7.95 ± 0.29; 2.36 ± 0.96 mg/ml vs. 1.16 ± 0.55 mg/ml; and 0.60 ± 0.21 vs. 0.31 ± 0.14, respectively). **f** The iodine-to-CT_VNC_ values of lipid-rich and lipid-poor adenomas were significantly different from those of metastases (12.31 ± 13.03 and 6.83 ± 2.65 vs. 3.33 ± 1.62, respectively). *Represents a significant difference in spectral parameters between lipid-rich adenomas and metastases (*p* < 0.05). ^#^Represents a significant difference in spectral parameters between lipid-poor adenomas and metastases (*p* < 0.05)

In the venous phase, the CT_VNC_ values of lipid-rich adenomas were lower than those of metastases (*p* < 0.001). The ID, NID, s-SHC and Z-eff values of lipid-poor adenomas were higher than those of metastases (*p* < 0.05). The iodine-to-CT_VNC_ values of lipid-rich and lipid-poor adenomas significantly differed from those of metastases (*p* < 0.05). Box and whisker plots of the spectral parameters of lipid-rich adenomas, lipid-poor adenomas and metastases in the venous phase are shown in Fig. 4.

### Differential diagnostic value of spectral parameters in adenomas and metastases

**Fig. 5:**
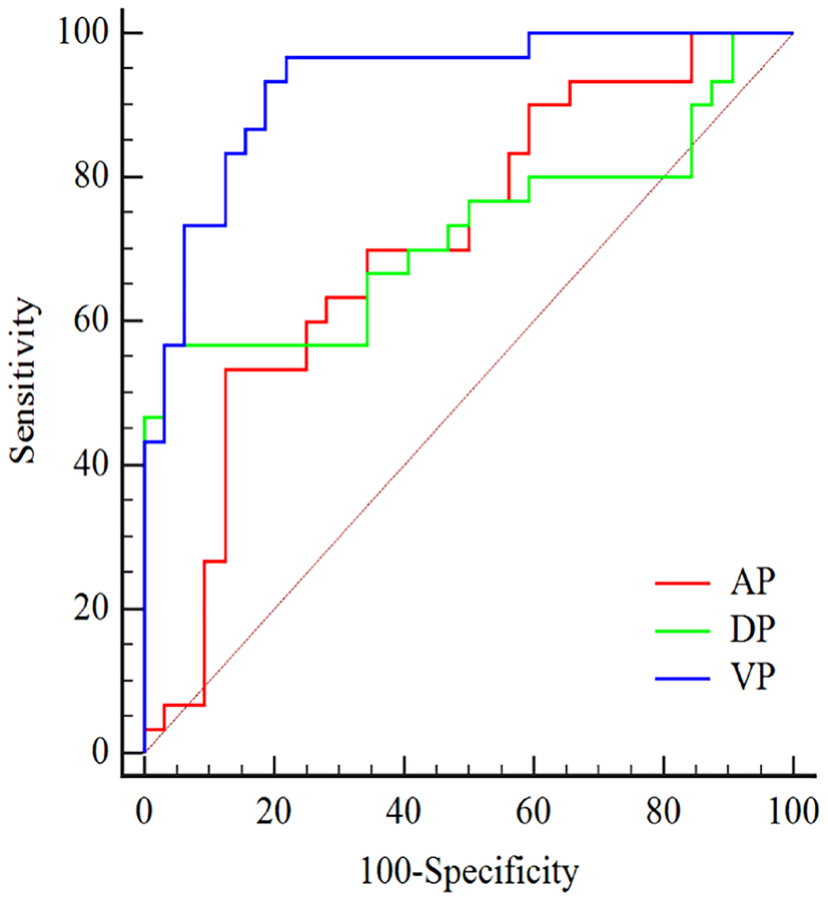
ROC curves for the combined spectral parameters (CT_VNC_ value + s-SHC value + Z-eff value + ID value) in the differential diagnosis of adrenal adenomas and metastases in the arterial phase (AP), venous phase (VP) and delayed phase (DP). The AUC of the combined spectral parameters in the venous phase was higher than that in the arterial and delayed phases (0.928 vs. 0.723 vs. 0.709, *p* < 0.05)

The combined spectral parameters (CT_VNC_ value + s-SHC value + Z-eff value + ID value) showed a better diagnostic performance in the venous phase than in the arterial or delayed phase (*p* < 0.05), with an AUC of 0.928 (95% CI 0.833–0.978). The ROC curves for the combined spectral parameters in the differential diagnosis of adenomas and metastases in different phases are shown in Fig. 5.

In the venous phase, the AUC of the iodine-to-CT_VNC_ value was higher than those of other spectral parameters in the differential diagnosis of adenomas and metastases (*p* < 0.05). The diagnostic sensitivity and specificity of the iodine-to-CT_VNC_ ratio were 74.4% and 91.9%, respectively.

The AUCs for CT_VNC_ and iodine-to-CT_VNC_ values were higher than those of other spectral parameters in the differential diagnosis of lipid-rich adenomas and metastases. The AUC for the CT_VNC_ value was 0.966. The diagnostic sensitivity and specificity of the CT_VNC_ value were 97.7% and 91.2%, respectively. The AUC for the iodine-to-CT_VNC_ ratio was 0.945. The diagnostic sensitivity and specificity of the iodine-to-CT_VNC_ value were 100% and 72.7%, respectively.

The AUCs of s-SHC and NID values were higher than those of other spectral parameters in the differential diagnosis of lipid-poor adenomas and metastases. The AUC for the s-SHC value was 0.920. The diagnostic sensitivity and specificity of the s-SHC value were 79.1% and 93.1%, respectively. The AUC for the NID value was 0.905. The diagnostic sensitivity and specificity of the NID value were 72.1% and 100%, respectively.

The AUC, sensitivity, and specificity values for each spectral parameter in the differential diagnosis of adenomas and metastases are shown in Table 3.

**Table 3:** The diagnostic AUC, sensitivity and specificity for spectral parameters in the differential diagnosis of adenomas and metastases

	Spectral parameters	Threshold	AUC^#^	sensitivity%^#^	specificity%^#^	Youden’s index
Adenomas vs metastases	ID/CT_VNC_	4.18	0.920 (0.840–0.958)	74.42 (58.8–86.5)	91.94 (82.2–97.3)	0.66
	CT_VNC_ (HU)	23	0.743 (0.649–0.823)	100 (91.8–100)	52.38 (39.4–65.1)	0.52
	s-SHC	2.1	0.723 (0.628–0.805)	95.35 (84.2–99.4)	42.86 (30.5––56.0)	0.38
	Z-eff	8.25	0.706 (0.610–0.791)	93.02 (80.9–98.5)	42.86 (30.5–56.0)	0.36
	ID (mg/ml)	1.53	0.695 (0.598–0.781)	81.4 (58.8–86.5)	50.79 (37.9––63.6)	0.32
	NID	0.4	0.668 (0.570–0.757)	74.42 (58.8–86.5)	53.97 (40.9–66.6)	0.28
Lipid-rich adenomas vs metastases	CT_VNC_ (HU)	24.13	0.966 (0.897–0.994)	97.67 (87.7–99.9)	91.18 (76.3–98.1)	0.89
	ID/CT_VNC_	6.93	0.945 (0.868–0.984)	100 (91.8–100)	72.73 (54.5–86.7)	0.73
	s-SHC	1.15	0.555 (0.437–0.668)	41.86 (27.0–57.9)	76.47 (58.8–89.3)	0.18
	Z-eff	7.85	0.544 (0.426–0.658)	41.86 (27.0–57.9)	76.47 (58.8–89.3)	0.18
	NID	0.17	0.534 (0.417–0.648)	79.07 (64.0–90.0)	0 (0.0–10.3)	0.21
	ID (mg/ml)	0.93	0.524 (0.407–0.639)	37.21(23.0–53.3)	76.47(58.8–89.3)	0.14
Lipid-poor adenomas vs metastases	s-SHC	1.73	0.920 (0.832–0.971)	79.07 (64.0–90.0)	93.1 (77.2–99.2)	0.72
	NID	0.39	0.905 (0.813–0.962)	72.09 (56.3–84.7)	100 (88.1–100.0)	0.72
	Z-eff	8.16	0.897 (0.802–0.956)	83.72 (69.3–93.2)	82.76 (64.2–94.2)	0.66
	ID (mg/ml)	1.53	0.895 (0.801–0.955)	81.4 (66.6–91.6)	82.76 (64.2–94.2)	0.64
	ID/CT_VNC_	4.18	0.892 (0.796–0.953)	74.42 (58.8–86.5)	89.66 (72.6–97.8)	0.64
	CT_VNC_ (HU)	40.75	0.518 (0.397–0.637)	83.72 (69.3–93.2)	34.48 (17.9–54.3)	0.18

*CT*_*VNC*_ CT value of virtual non-contrast images, *s-SHC* slope of spectral HU curve, *Z-eff* Z-effective, *ID* iodine density, *NID* normalized iodine density, *ID/CT*_*VNC*_ iodine-to-CT_VNC_

^#^With 95% confidence intervals in brackets. Youden’s index = sensitivity + specificity − 1

## Discussion

In the adrenal glands, adenomas and metastases are the most common benign and malignant tumors, respectively. In some cases such as patients with primary malignant tumors, identification of the incidentalomas’ biological characteristics is somewhat challenging. However, various spectral parameters generated by spectral imaging based on dual-energy CT systems may provide more helpful clues to tackle this clinical problem. Our results revealed that all spectral parameters were significantly different between adenomas and metastases in the venous phase (all *p* < 0.05). The combined spectral parameters (CT_VNC_ value + s-SHC value + Z-eff value + ID value) showed a better diagnostic performance in the venous phase than in the arterial or delayed phase (all *p* < 0.05). The AUCs of iodine-to-CT_VNC_, CT_VNC_ and s-SHC values were higher than those of other spectral parameters in the venous phase in differentiating adenomas, lipid-rich adenomas and lipid-poor adenomas from metastases, respectively.

In contrast to enhanced CT scanning, adrenal adenomas typically display fast wash-in and rapid washout, while metastases reveal slow wash-in and relatively sustained enhancement (Mayo-Smith et al. 2017). Wash-out scan of the adrenal commonly performs 10 or 15 min after contrast injection requiring an additional patient’s separate visit of CT scan. Several studies have shown the wash-out threshold of adenomas differing with the time of the delayed phase scan (Ng et al. 2018; Liu et al. 2019; Botsikas et al. 2014). Our study started delayed phase scanning on a short-time of 180 s after contrast injection, which was similar to Ng’s and Liu’s study (Ng et al. 2018; Liu et al. 2019). They started delayed phase scanning on 120–248 s and 200 s, respectively. The diagnostic performance of RPW was better than APW in the difference between adrenal benign and malignant tumors, in accordance with previous studies. The 21% criterion of RPW for adenomas yielded 94% sensitivity and 90% specificity, while for lipid-rich adenomas yielded 100% sensitivity and 90% specificity.

There are multiple phases in enhanced adrenal CT examinations, but few studies have analyzed the diagnostic performances of spectral parameters in different scanning phases to differentiate adenomas from metastases. The above results indicated that all spectral parameters in the venous phase (CT_VNC_ value, s-SHC value, Z-eff value, ID value, NID value and iodine-to-CT_VNC_ value) were significantly different between adenomas and metastases. Furthermore, the diagnostic performance of the combined spectral parameters in the venous phase was superior to that in the arterial or delayed phase (*p* < 0.05).

VNC images are reconstructed by postprocessing spectral CT scans by removing iodine from tumors, tissues and vessels, thus generating images similar to true non-contrast (TNC) images (D’Angelo et al. 2021). Connolly et al. (2017) pointed out that the sensitivity and specificity of the CT_VNC_ value in the diagnosis of adrenal adenoma are relatively low, i.e., 54% and 57%, respectively. To improve the diagnostic value, a study by Nagayama et al. (2020) employed the iodine-to-CT_VNC_ ratio for the differential diagnosis of adrenal adenomas and metastases, and the AUC of the iodine-to-CT_VNC_ value was 0.98. The sensitivity and specificity of iodine-to-CT_VNC_ value were both 95%. Our results revealed that the AUC of the iodine-to-CT_VNC_ ratio was 0.920. With a threshold of 4.18, the sensitivity and specificity of the ratio were 92% and 74%, respectively. Differences in iodine density values for adrenal tumors may be responsible for the differences between Nagayama’s results (adenomas vs. metastases, 2.4 mg/mL vs. 1.7 mg/mL) and ours (adenomas vs. metastases, 1.75 mg/mL vs. 1.16 mg/mL). Differences in scanning protocols including the injection rate of the contrast agent and scanning phase in two studies may lead to differences in the iodine density of lesions.

The iodine density is one of the most commonly used spectral parameters and has shown promising results for the diagnoses of various research such as metastatic lymph node, pulmonary thromboembolism, pancreatitis, and adrenal tumors (Martin et al. 2019, 2018; Cicero et al. 2020; Mileto et al. 2015). Martin et al. (2018) showed that in the portal phase, the ID value of adenomas was significantly lower than that of metastases (1.3 ± 0.4 mg/ml vs. 3.2 ± 1.4 mg/ml, *p* < 0.001). Contrary to their study, our results revealed that the ID value of adenomas in the venous phase was higher than that of metastases (1.75 ± 0.93 mg/mL vs. 1.16 ± 0.55 mg/mL, *p* < 0.05). Reasons for the differences between the results of Martin’s study and ours may be as follows: (1) the majority of primary cancers in the metastasis group were renal carcinoma (12/26, 46.2%) in Martin’s study. Previous findings (Moosavi et al. 2016) suggested that lipids are present in adrenal metastases from renal carcinoma, decreasing the ability of the metastases to uptake iodine, which may lead to differences between the two studies. (2) Martin’s study started portal phase scanning 70 s after the contrast agent injection, while we used the operating mode of scan triggering. Individual variations in cardiac function and hemodynamic characteristics may be more obvious without scan triggering mode. (3) A dual-source CT (DSCT) system was employed in Martin’s study, while a DLSCT system was used in this study. The measurement error of iodine density is the difference between the iodine density measured in a phantom and that measured on images generated by the CT system. Phantom studies (Sellerer et al. 2018) revealed that this error for the DSCT system is far greater than that of the DLSCT system.

Virtual monoenergetic image (VMI) derived from dual-energy CT provide great diagnostic value (Albrecht et al. 2019). The s-SHC value can reflect the spectral curve shape of a specific tissue, and the slope of the 40–100-keV curve is usually employed. Previous studies proposed that the spectral curves of adenomas generally have an ascending or descending shape, while most curves of metastases have a descending shape in plain images (Gupta et al. 2010; Ju et al. 2015). Our study pointed out that s-SHC values of lipid-rich adenomas, lipid-poor adenomas and metastases were 0.71, 2.05 and 1.18, respectively. With a threshold of 4.18, the AUC of the s-SHC value (0.920) was higher than that of other parameters in distinguishing lipid-poor adenomas and metastases, and the diagnostic sensitivity and specificity of the s-SHC value were 79.1% and 93.1%, respectively.

There were a few limitations in this research. First, this was a single-center clinical trial with a relatively small sample size. Second, most metastatic lesions were diagnosed based on imaging evidence. This was similar to many previous studies of adrenal imaging (Kim et al. 2013; Martin et al. 2018; Laukamp et al. 2021). Third, the spectral characteristics of adrenal metastases from different primary malignant tumors have not been analyzed owing to the small sample size, and further research is warranted for a thorough investigation. Forth, only spectral parameters of adrenal adenomas and metastases were assessed in our research, further investigation of other incidentalomas including benign and malignant are needed.

In conclusion, on DLSCT, the combined spectral parameters in the venous phase are efficient in distinguishing adrenal adenomas from metastases. The iodine-to-CT_VNC_ ratio, CT_VNC_ value and s-SHC value (0.920, 0.966 and 0.920, respectively) had the highest AUCs in differentiating adenomas, lipid-rich adenomas and lipid-poor adenomas from metastases, respectively. Therefore, spectral imaging provides the morphological features, enhancement patterns, attenuations wash-out values, and spectral parameters of adrenal lesions, which is beneficial for the differential diagnoses of adrenal adenomas and metastases.

## Supplementary Information

Below is the link to the electronic supplementary material.

Supplementary file1 (DOCX 39 KB)

## Data Availability

The original contributions presented in the study are included in the article/supplementary material. And further inquiries can be directed to the corresponding author.
