# Supplementary material for: Differential diagnosis of adrenal adenomas and metastases using spectral parameters in dual-layer detector spectral CT
Source: J Cancer Res Clin Oncol. 2023 Jun 6;149(12):10453–63. doi: 10.1007/s00432-023-04931-8 (PMC10423139; doi:10.1007/s00432-023-04931-8)
Supplement: Supplementary file 1 — Supplementary file1 (DOCX 39 KB) [file 432_2023_4931_MOESM1_ESM.docx]

Supplementary Material

**Supplementary Table 1.** CT values and wash-out values of adrenal adenomas and metastases.

|  | CT-unenhanced | CT-AP | CT-VP | CT-DP | APW | RPW |
| --- | --- | --- | --- | --- | --- | --- |
| Adenomas | 9.79±18.16* | 48.61±32.81 | 63.54±33.41 | 22.61±29.46* | 133.85±244.46* | 50.61±29.07* |
| Lipid-rich adenomas | -4.64±8.65* | 25.11±17.77* | 40.61±18.07* | 7.88±11.83* | 165.65±145.01* | 63.89±28.74* |
| Lipid-poor adenomas | 26.71±9.64* | 76.16±23.56* | 90.42±26.45* | 38.98±34.53 | 103.83±312.68 | 38.05±23.86* |
| Metastases | 33.47±7.82 | 54.61±19.20 | 61.69±16.78 | 52.38±24.41 | 98.13±438.10 | 19.15±37.51 |
| Note: * represents a significant difference in parameters between adenomas, lipid-rich adenomas, and lipid-poor adenomas from metastases (P<0.05). Abbr: CT values on unenhanced phase (CT-unenhanced), CT values on arterial phase (CT-AP), CT values on venous phase (CT-VP), CT values on delayed phase (CT-DP), absolute percentage of enhancement washout (APW), relative percentage of enhancement washout (RPW). | | | | | | |

**Supplementary Table 2.** The diagnostic AUC, sensitivity and specificity for CT values and wash-out values in the differential diagnosis of adenomas and metastases.

|  | Parameters | Threshold | AUC* | sensitivity%* | specificity%* | Youden’s index |
| --- | --- | --- | --- | --- | --- | --- |
| Adenomas VS Metastases | CT-unenhanced | 16.77 | 0.862 (0.782～0.921) | 65.08 (52.0～76.7) | 97.67 (87.7～99.9) | 0.63 |
|  | RPW % | 20.99 | 0.837 (0.732～0.913) | 94.29 (80.8～99.3) | 78.95 (62.7～90.3) | 0.73 |
|  | APW % | 38.69 | 0.672 (0.552～0.778) | 82.86 (66.4～93.4) | 63.16 (46.0～78.2) | 0.46 |
|  | CT-DP | 40 | 0.672 (0.541～0.786) | 50 (31.3～68.7) | 96.87 (83.8～99.9) | 0.47 |
|  | CT-AP | 34.75 | 0.580 (0.480～0.675) | 44.44 (31.9～57.5) | 90.70 (77.9～97.4) | 0.35 |
|  | CT-VP | 44.7 | 0.517 (0.418～0.615) | 38.10 (26.1～51.2) | 86.05 (72.1～94.7) | 0.24 |
| Lipid-rich adenomas VS Metastases | CT-unenhanced | 8.77 | 1.000 (0.953～1.000) | 100 (89.7～100) | 100 (91.8～100.0) | 1 |
|  | CT-DP | 30.2 | 0.998 (0.919～1.000) | 30.2 (76.8～100.0) | 96.87 (83.8～99.9) | 0.97 |
|  | CT-AP | 39.35 | 0.882 (0.788～0.944) | 85.26 (68.9～95.0) | 83.72 (69.3～93.2) | 0.69 |
|  | RPW % | 20.99 | 0.859 (0.739～0.938) | 100 (80.5～100) | 78.95 (62.7～90.4) | 0.79 |
|  | CT-VP | 53.05 | 0.813 (0.708～0.893) | 82.35 (65.5～93.2) | 76.74 (61.4～88.2) | 0.59 |
|  | APW % | 38.69 | 0.765 (0.631～0.869) | 100 (80.5～100) | 63.16 (46.0～78.2) | 0.63 |
| Lipid-poor adenomas VS Metastases | CT-VP | 69.15 | 0.830 (0.724～0.909) | 75.86 (56.6～89.7) | 76.74 (61.4～88.2) | 0.53 |
|  | RPW % | 23.36 | 0.816 (0.690～0.907) | 88.89 (65.3～98.6) | 81.58 (65.7～92.3) | 0.7 |
|  | CT-AP | 68 | 0.775 (0.661～0.865) | 72.41 (52.8～87.3) | 88.37 (74.9～96.1) | 0.61 |
|  | CT-unenhanced | 36.33 | 0.700 (0.581～0.803) | 89.66 (72.6～97.8) | 44.19 (29.1～60.1) | 0.34 |
|  | CT-DP | 59.93 | 0.612 (0.461～0.749) | 75 (0.445～0.715) | 53.13 (34.7～70.9) | 0.28 |
|  | APW % | 55.19 | 0.585 (0.445～0.715) | 61.11 (35.7～82.7) | 71.05 (54.1～84.6) | 0.32 |
| Note: * with 95% confidence intervals in brackets. Youden’s index=sensitivity+specificity-1. Abbr: CT values on unenhanced phase (CT-unenhanced), CT values on arterial phase (CT-AP), CT values on venous phase (CT-VP), CT values on delayed phase (CT-DP), absolute percentage of enhancement washout (APW), relative percentage of enhancement washout (RPW). | | | | | | |

**Supplementary Table 3.** The ICC values of spectral parameters in adrenal adenomas and metastases.

|  | **Group** | **CT_VNC_*** | **s-SHC*** | **Z-eff*** | **ID*** |
| --- | --- | --- | --- | --- | --- |
|  |  |  |  |  |  |
| **Arterial phase** | Adenomas | 0.971 (0.953～0.983) | 0.948 (0.916～0.968) | 0.967 (0.947～0.980) | 0.991 (0.985～0.994) |
|  | Lipid-rich | 0.946 (0.895～0.973) | 0.876 (0.766～0.936) | 0.920 (0.846～0.959) | 0.969 (0.939～0.984) |
|  | Lipid-poor | 0.876 (0.753～0.940) | 0.928 (0.852～0.965) | 0.965 (0.927～0.984) | 0.990 (0.979～0.995) |
|  | Metastases | 0.877 (0.801～0.937) | 0.929 (0.872～0.961) | 0.959 (0.925～0.977) | 0.987 (0.976～0.993) |
| **Venous phase** | Adenomas | 0.974 (0.958～0.984) | 0.958 (0.932～0.975) | 0.968 (0.947～0.980) | 0.991 (0.986～0.995) |
|  | Lipid-rich | 0.954 (0.911～0.977) | 0.869 (0.754～0.932) | 0.920 (0.846～0.959) | 0.971 (0.943～0.985) |
|  | Lipid-poor | 0.910 (0.817～0.957) | 0.956 (0.908～0.979) | 0.962 (0.921～0.982) | 0.991 (0.981～0.996) |
|  | Metastases | 0.869 (0.769～0.926) | 0.874 (0.780～0.930) | 0.920 (0.857～0.956) | 0.976 (0.955～0.987) |
| **Delayed phase** | Adenomas | 0.986 (0.970～0.993) | 0.959 (0.916～0.980) | 0.983 (0.964～0.992) | 0.985 (0.968～0.993) |
|  | Lipid-rich | 0.944 (0.835～0.982) | 0.927 (0.789～0.976) | 0.930 (0.797～0.977) | 0.973 (0.918～0.991) |
|  | Lipid-poor | 0.906 (0.753～0.966) | 0.909 (0.760～0.967) | 0.974 (0.926～0.991) | 0.959 (0.886～0.985) |
|  | Metastases | 0.874 (0.757～0.936) | 0.878 (0.764～0.938) | 0.888 (0.783～0.944) | 0.951 (0.902～0.976) |
| Note: * with 95% confidence intervals in brackets. ICC: 0.75～1.00 (excellent), 0.60～0.74 (good), 0.40～0.59 (fair), 0.00～0.39 (poor). Abbr: CT value of virtual non-contrast images (CT_VNC_), slope of spectral HU curve (s-SHC), Z-effective (Z-eff), iodine density (ID). | | | | | |
